# CAMKIIγ is a targetable driver of multiple myeloma through CaMKIIγ/ Stat3 axis

**DOI:** 10.18632/aging.103490

**Published:** 2020-07-13

**Authors:** Linlin Yang, Bowen Wu, Zhaoxing Wu, Ying Xu, Ping Wang, Mengyuan Li, Rongzhen Xu, Yun Liang

**Affiliations:** 1 Department of Hematology, Key Laboratory of Cancer Prevention and Intervention, China National Ministry of Education, Key Laboratory of Molecular Biology in Medical Sciences, Zhejiang Province, The Second Affiliated Hospital, College of Medicine, Zhejiang University, Hangzhou 310000, Zhejiang, China; 2 Cancer Institute of Zhejiang University, Hangzhou 310000, Zhejiang, China

**Keywords:** CaMKIIγ, multiple myeloma, Stat3, poor prognosis

## Abstract

Aberrant activation of CAMKIIγ has been linked to leukemia and T-cell lymphoma, but not multiple myeloma (MM). The purpose of this study was to explore the role of CaMKIIγ in the pathogenesis and therapy of MM. In this study, we found that CaMKIIγ was aberrantly activated in human MM and its expression level was positively correlated with malignant progression and poor prognosis. Ectopic expression of CaMKIIγ promoted cell growth, colony formation, cell cycle progress and inhibited apoptosis of MM cell lines, whereas, knockdown of CAMKIIγ expression suppressed MM cell growth in vitro and in vivo. Mechanically, we observed that CaMKIIγ overexpression upregulated p-ERK and p-Stat3 levels and suppression of CaMKIIγ had opposite effects. CaMKIIγ is frequently dysregulated in MM and plays a critical role in maintaining MM cell growth through upregulating STAT3 signaling pathway. Furthermore, our preclinical studies suggest that CaMKIIγ is a potential therapeutic target in MM, and could be intervened pharmacologically by small-molecule berbamine analogues.

## INTRODUCTION

Multiple myeloma (MM) is a malignant hematologic disease characterized by clonal proliferation of malignant plasma cells in the bone marrow [1]. In recent years, survival of MM patients has at least doubled, due to the applications of proteasome inhibitors (PI), immunomodulatory agents (IMiDs), monoclonal antibodies and stem-cell transplantation [2–4]. However, prognosis of MM still remains unsatisfactory with most patients experiencing drug resistance or early relapse [5]. It is necessary to explore more potent and safer therapies against novel MM targets.

Ca2+/calmodulin–dependent protein kinase II (CAMKII), multifunctional serine/threonine kinases, is a crucial mediator of translating intracellular Ca^2+^ signals into cellular responses [6]. It has 4 different isoforms with distinct expression patterns, indicating that isoform-specific interventions might be relatively tissue selective in mammalian cells [7]. Our previous studies demonstrated that CAMKIIγ is aberrantly activated and plays critical roles in the blast crisis progression of chronic myeloid leukemia [8]. Moreover, we found that CAMKIIγ is a critical regulator of MYC protein stability in human T-cell lymphoma [9]. Interestingly, CAMKIIγ is a critical regulator of multiple oncogenetic signaling pathways such as JAK2/STAT3 [10, 11] that play important roles in MM pathogenesis [12]. In addition, we demonstrated that the natural compound berbamine (BBM) is a CAMKIIγ inhibitor and potently inhibits T-cell lymphoma growth in mouse model [9]. However, it is unknown whether CAMKIIγ plays a role in MM and whether BBM could inhibit the growth of MM cell in mouse models.

In this study, we first evaluated whether CAMKIIγ is dysregulated in MM patients, and how it affects MM cell function if so, we then systemically investigated the effects of berbamine and its derivative WBC158 on the growth of MM cells in vitro and in vivo. We demonstrated that CAMKIIγ was aberrantly activated in majority of MM patients, and associated with poor prognosis. Both in vitro and in vivo studies revealed that CAMKIIγ is essential for MM cell growth and berbamine analogues potently suppressed the growth of MM cells via targeting CAMKIIγ/STAT3 axis.

## RESULTS

### CAMKIIγ overexpression is correlated with malignant progression and poorer clinical outcomes of MM patients

**Figure 1:**
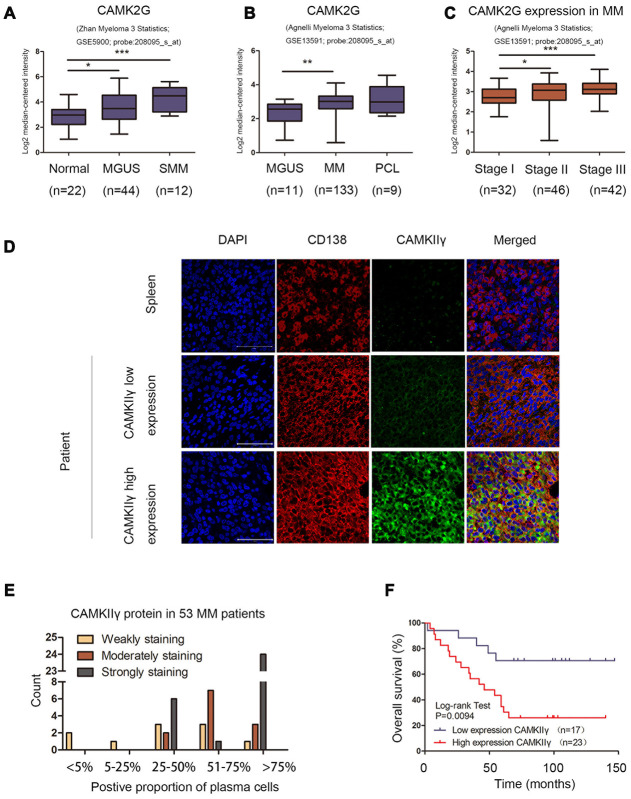
**Overexpression of CAMKIIγ was associated with disease progression and poor prognosis of MM patients.** (**A**, **B**) CAMK2G was differentially expressed in samples from healthy donors, monoclonal gammopathy of undetermined significance (MGUS), smoldering MM (SMM), MM, or PCL patients in the indicated datasets (*P < 0.05, **P < 0.01, ***P < 0.001). (**C**) The distribution of CAMK2G mRNA was at different stage of MM (*P < 0.05, **P < 0.01, ***P < 0.001). (**D**) CAMKIIγ protein expression was measured by immunofluorescent trichrome staining of DAPI (blue nuclear staining), plasma cell marker CD138 (red membrane staining) and CAMKIIγ (green cytoplasm staining). Representative images were shown at 630 X magnification. The white scale bar represented 50 μm. (**E**) The distribution of CAMKIIγ protein in 53 MM patients analyzed. (**F**) Kaplan-Meier overall survival curve for CAMKIIγ expression in 40 MM patients with extramedullary disease. Patients with high CAMKIIγ expression were significantly associated with poorer overall survival (*P* = 0.0094).

To evaluate the clinical significance of CAMK2G in MM pathogenesis, we examined two independent microarray datasets from GEO databases (GSE5900 [13, 14], GSE13591 [15]) and found that elevated CAMK2G mRNA was associated significantly with disease progression from normal or clinically insidious stages to malignant MM (Figure 1A, 1B). In patients with MM, by querying GSE13591 data set, we found that CAMK2G was significantly upregulated at advanced stage, compared to stage I (Figure 1C). We then analyzed CAMKIIγ protein expression level in paraffin slices of extramedullary infiltrating tissues from MM patients (n = 53) and normal tissues containing plasma cells (n = 4) using immunohistochemical staining. The results of normal samples showed that three tissues were weakly staining in less than 10% of the plasma cells, and one tissue with no staining. In contrast, CAMKIIγ protein among MM samples displayed higher expression than normal tissues and the distribution was shown in Figure 1E. Thirty-six specimens exhibited medium to strong CAMKIIγ expression in more than 50% of the plasma cells. Two samples showed < 25% CAMKIIγ-positive plasma cells and the remaining MM samples were stained weakly in more than 25% of the cells. Representative samples of MM patients were co-stained with DAPI, CAMKIIγ and the plasma cell marker CD138 by immunofluorescent staining to further comfirm above results (Figure 1D). In conclusion, the presence of CAMKIIγ protein was detected in all of patient samples analyzed, and CAMKIIγ protein expression was highly expressed in 29 of the 53 (54.7%) MM patients examined.

Next, we analyzed the relationship between CAMKIIγ expression and clinic pathological features of MM patients. It should be clearly noted that our cohort contained too few cases with FISH defined high-risk cytogenetics to allow correlative analysis. As summarized in Table 1, high CAMKIIγ expression had a significant association with DS stage III (*P* = 0.037) and the number of bone lesions (≥ 3) (*P* = 0.031). However, there were no statistically significant between CAMKIIγ expression and the remaining factors.

**Table 1:** Clinicopathological features of MM patients according to high and low CAMKIIγ expression.

**Clinicopathological features**	**Low CAMKIIγ (n = 24)**	**High CAMKIIγ (n = 29)**	***P* value**
Age (yr), median (range)	56.5 (32-77)	60 (41-81)	0.221^a^
Sex, female/male (%/%)	11/13 (45.8/54.2)	10/19 (34.5/65.5)	0.400^b^
DS stage number (%)			
I and II	16 (66.7)	11 (37.9)	0.037^b^
III	8 (33.3)	18 (62.1)	
ISS stage number (%)			
I and II	20 (83.3)	25 (92.6)	0.402^b^
III	4 (16.7)	2 (7.4)	
NA^c^	0	2	
Immunoglobulin subtype number (%)
IgG	10 (45.5)	7 (26.9)	0.222^b^
IgA	5 (22.7)	10 (38.5)	
IgM	0	0	
IgD	0	1 (3.8)	
Light-chain only	5 (22.7)	8 (30.8)	
Non-secretory	2 (9.1)	0	
NA^c^	2	4	
Albumin (g/L), median (range)	36.95 (20.1-48.3)	36.3 (20.1-50.8)	0.950^a^
Serum creatinine (mg/dL), median (range)	69.84 (31-176)	61.00 (28.29-171.50)	0.655^a^
Hemoglobin (g/L), median (range)	112 (76-157)	113 (59-157)	0.986^a^
β2-microglobulin (mg/L)
Median (range), NA	2.45 (1.02-14.56), 0	2.68 (1.48-10.27), 3	0.497^a^
Serum LDH (IU/L), median (range)	161.5 (72-981)	61.00 (28.29-171.50)	0.858^a^
Number of bone lesions (%)			
<3	16 (69.6)	11 (39.3)	0.031^b^
≥3	7 (30.4)	17 (60.7)	
NA	1	1	

^a^Mann-Whitney U test; ^b^Chi-square test; ^c^Not available

The bold number shows the *P*-values with significant differences. *P* < 0.05 represents statistically significant difference.

The results of Kaplan-Meier analysis and the Log-rank test indicated that patients with high CAMKIIγ expression (n = 23) were significantly associated with poor overall survive (OS) than patients with low CAMKIIγ expression (n = 17) (*P* < 0.0094, Figure 1F). CAMKIIγ expression, age, LDH, β_2_-MG, DS stage and ISS stage were analyzed using univariate and multivariate Cox regression analyses (Table 2). In this model, LDH lost prognostic significance. Cox regression survival analysis incorporating age, β2-MG, DS and ISS stage, high CAMKIIγ expression was an independent prognostic indicator in MM patients with a hazard ratio of 4.251. In addition, age (> 65), β2-MG (≥ 5.5mg/L), DS stage III and ISS stage III also showed significant association with inferior OS.

**Table 2:** Univarite and multivariate Cox regression analyses of prognostic parameters for overall survival in MM patients.

**Prognostic parameter**	**Univariate analysis**			**Multivariate analysis**		
	**HR^a^**	**95%CI^b^**	***P* value**	**HR**	**95%CI**	**P value**
CAMKIIγ (low vs.high)	3.472	1.276-9.448	**0.015**	4.251	1.369-13.194	**0.012**
Age (≤65 vs >65)	2.526	1.074-5.937	**0.034**	5.077	1.793-14.372	**0.002**
LDH (≤271 vs >271)	3.33	0.954-11.624	0.059	-	-	-
β2-MG (<5.5 vs ≥5.5)	4.038	1.292-12.619	**0.016**	4.109	1.099-15.367	**0.036**
DS stage (I/ II vs III)	4.666	1.797-12.114	**0.002**	4.576	1.461-14.331	**0.009**
ISS stage (I/ II vs III)	4.188	1.339-13.092	**0.014**	4.109	1.099-15.367	**0.036**

^a^HR: Hazard ratio; ^b^CI: Confidence interval. The bold number represents the *P*-value with significant differences.

Collectively, these observations indicate a role of overexpressed CAMKIIγ in promoting malignant progression and poor clinical outcomes of MM patients.

### CAMKIIγ is crucial for MM cell proliferation and colony formation in vitro

**Figure 2:**
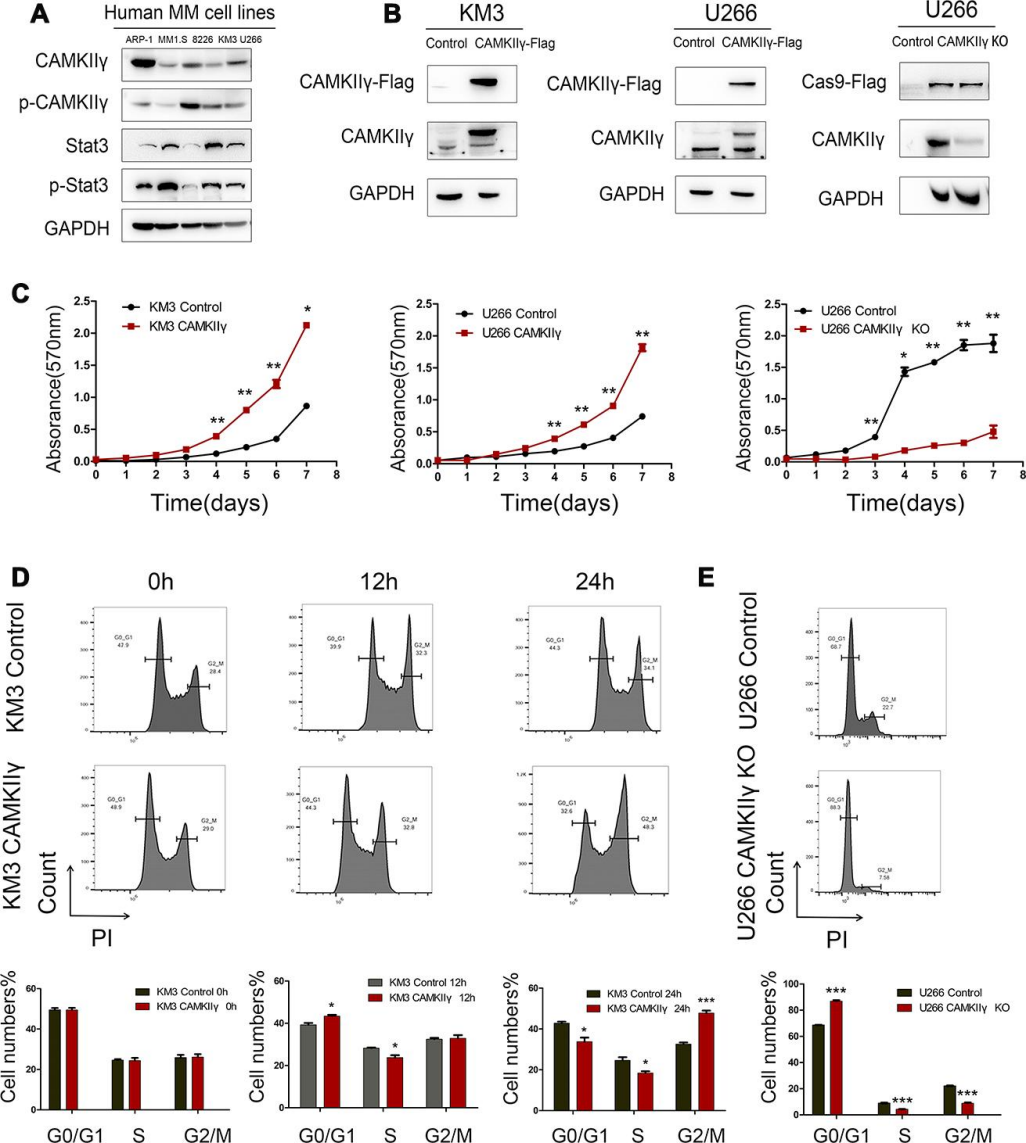
**Effects of CAMKIIγ on proliferation and cycle progression of MM cells.** (**A**) CAMKIIγ and Stat3 protein expression in human MM cell lines, including ARP-1, MM1.S, 8226, KM3, U266 cell lines. (**B**) CAMKIIγ overexpression or knockout efficiency was measured by Western blot. GAPDH was used as a loading control. (**C**) Proliferation curves of KM3 or U266 stable-transfected cells with CAMKIIγ overexpression or knockout, compared with their controls. The MTT assays were performed for a total of a week (*P < 0.05, **P < 0.01). (**D**) KM3 cells of CAMKIIγ overexpression and the control were cultured in serum-free medium for 48 hours, then maintained in 1640 medium supplemented with 15% fetal bovine serum at the indicated times. Representative images and quantification of cell cycle by flow cytometry (*P < 0.05, ***P < 0.001). (**E**) Representative images and quantification of cell cycle in U266 cells after DOX-induced CAMKIIγ-KO (***P < 0.001).

**Figure 3:**
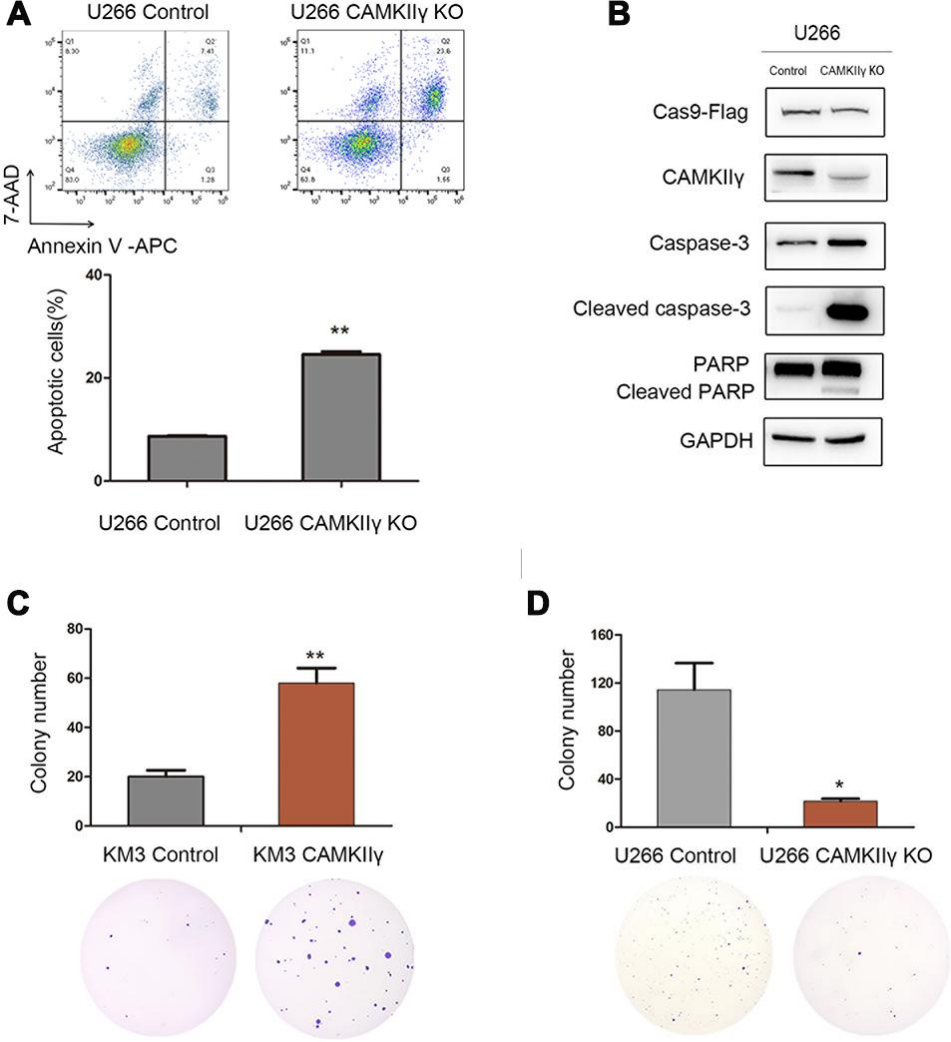
**CaMKIIγ was critically required for apoptosis and colony-forming of MM Cells.** (**A**) Representative images and quantification of apoptosis in U266 cells after DOX-induced CAMKIIγ-KO (**P < 0.01). (**B**) Expression levels of apoptosis-related protein were obviously increased in U266 cells of CaMKIIγ downregulation. Comparison of colony-forming ability of high CaMKIIγ expression (**C**, **P < 0.01), low CaMKIIγ expression (**D**, *P < 0.05) and the controls, respectively. Cells were plated in the growth medium in 6-well plates and the colonies were counted under light microscope after roughly 3 weeks.

The results of Western blot showed the expression profiles of CAMKIIγ protein in five human MM cell lines (Figure 2A). KM3 and U266 were used to construct stable-transfected cells of CAMKIIγ overexpression (KM3-CAMKIIγ and U266-CAMKIIγ) and corresponding controls. Meanwhile, the CAMKIIγ-dependent MM cell proliferation was confirmed by CRISPR/CAS9-mediated CAMKIIγ knockout (U266 CAMKIIγ KO; U266 control). CAMKIIγ overexpression or knockout efficiency was measured by Western blot (Figure 2B). In order to study the effects of CAMKIIγ on MM cell viability and proliferation ability, we performed MTT assay and colony formation assay. The MTT assays exhibited that ectopic expression of CAMKIIγ markedly enhanced cell proliferation as compared with the controls (Figure 2C, left and middle), whereas the downregulation of CAMKIIγ suppressed cell growth (Figure 2C, right). Additionally, we found high CAMKIIγ expression promoted colony formation (Figure 3C) and the colony-forming capacity was inhibited in CAMKIIγ knockout group (Figure 3D). Furthermore, CAMKIIγ depletion displayed G0/1 phase arrest and G2/M phase decrease (Figure 2E). On the contrary, CAMKIIγ overexpression promote cell cycle of MM cells (Figure 2D). Importantly, CAMKIIγ knockout led to a significant increase of cell apoptosis in U266 cells (Figure 3A) with a concomitant increase of cleaved Caspase-3 and cleaved PARP (Figure 3B).

These results show that CAMKIIγ is essential for proliferation, cell cycle progression and anti-apoptosis of MM cells in vitro.

### CAMKIIγ is essential for the growth of MM cells in vivo

**Figure 4:**
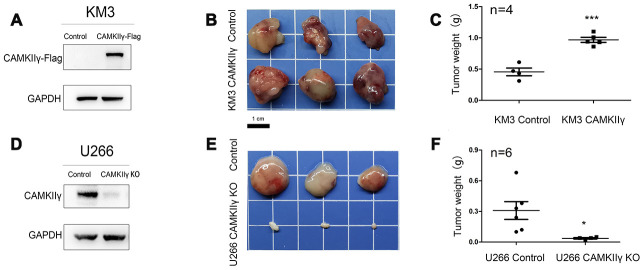
**CaMKIIγ was essential for MM tumorigenesis in vivo.** (**A**, **D**) CaMKIIγ overexpression or knockout was validated by Western blot. (**B**, **E**) Representative images of xenograft tumors from the indicated groups. BALB/C nude mice were injected subcutaneously in the left groin with 1.5x10^7 KM3 or U266 cells. Tumor blocks were removed from the mice at 3 weeks after inoculation. (**C**, **F**) Tumor weight in indicated groups (*P < 0.05, ***P < 0.001).

To determine whether CAMKIIγ was essential for MM tumorigenesis in vivo, we generated xenograft tumor models by subcutaneously injecting KM3 transfected and U266 transfected cells into the left groin of nude mice (Figure 4A, 4D). CAMKIIγ knockout was achieved by DOX induction. Tumor growth was obviously suppressed and the average tumor weight was significantly decreased in the group of CAMKIIγ downregulation compared to the control group (*P* < 0.05, Figure 4E, 4F). While CAMKIIγ overexpression significantly promoted the tumor growth of MM (*P* < 0.001, Figure 4B, 4C). As a result, the xenograft tumor models indicate CAMKIIγ is critically required for MM tumor growth in vivo.

### CAMKIIγ upregulates ERK/Stat3 signaling pathway of MM cells

Previous studies showed that CAMKIIγ promotes cellular survival and proliferation through MAPK/ERK and JAK2/Stat3 signaling pathways, which play important roles in the pathogenesis of MM [16–18]. These data prompted us to hypothesize that CAMKIIγ may also play a crucial role in regulating these signaling pathways in MM. Meanwhile, we investigated the expression profile of Stat3 in MM using five human MM cell lines by Western blot (Figure 2A).

**Figure 5:**
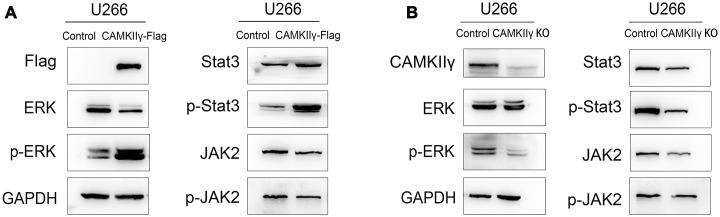
**CaMKIIγ regulated ERK and Stat3 in MM cells.** Expression of total-ERK, phospho-ERK, total-Jak2, phospho-Jak2, total-Stat3, phospho-Stat3 proteins were determined in U266 transfected cells of CaMKIIγ high-expression (**A**) and low-expression (**B**).

To test it, we first analyzed the levels of total and phosphorylated ERK, Stat3 and Jak2 in U266 cells with CAMKIIγ overexpression using Western blot. We observed that the levels of phosphorylated ERK and Stat3 were markedly increased, but total levels of ERK and Stat3 did not alter as compared with controls (Figure 5A, 5B). We next analyzed the levels of total and phosphorylated ERK and Stat3 in U266 cells with CAMKIIγ knock-down using western blot. We found that knockdown of CAMKIIγ reduced the levels of phosphorylated ERK and Stat3, but not total levels of ERK and Stat3. These results suggest CAMKIIγ plays critical roles in maintaining the activation of MAPK/ERK and JAK2/STAT3 signaling pathways of MM.

### Nature compound berbamine (BBM) and its analogue potently inhibited MM cell growth

**Figure 6:**
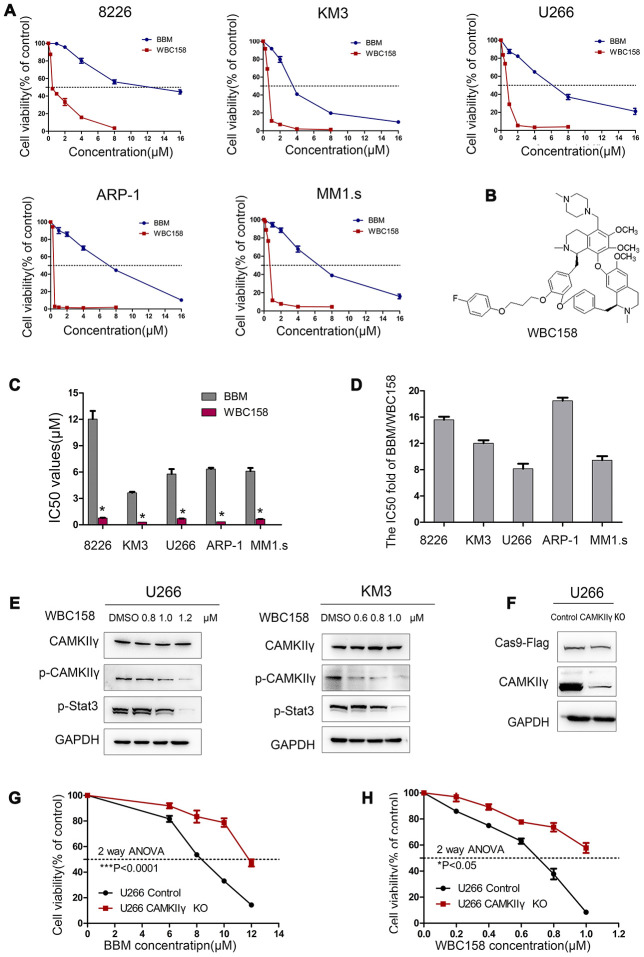
**WBC158 exhibited more potent anti-proliferation activity than BBM partially through targeting CaMKIIγ.** (**A**) MM cells were treated with the indicated concentrations of BBM and WBC158 for 72 h. (**B**) The chemical structure of WBC158. (**C**) Comparison of IC50 values of BBM and WBC158 in MM cells (*P < 0.05). (**D**) The IC50 value fold of BBM and WBC158. (**E**) KM3 or U266 cells was treated with WBC158 for 24 h. CaMKIIγ, p-CaMKIIγ and p-Stat3 proteins expression were detected by Western blot. (**F**) Western blot of U266 cells after DOX-induced CAMKIIγ -KO. CAMKIIγ knockout and the control in U266 cells were treated with BBM (**G**) or WBC158 (**H**) at various concentrations for 72h (*P < 0.05, ***P < 0.001).

Our previous study showed that BBM exerted its anti-tumor effects by blocking the ATP binding pocket of CAMKIIγ, and BBM analogues could inhibit MM cell growth [19–22]. WBC158, a BBM analogue, was used to evaluate its effects on the growth and CAMKIIγ activity. The structure of WBC158 was shown in Figure 6B. Five MM cell lines were separately treated with BBM and WBC158 at the indicated concentrations for 72 h and then collected for analyses of cell variability. As a result, WBC158 exhibited more potent anti-proliferation activity than BBM (Figure 6A, 6C). Compared with that of BBM, WBC158 exhibited 15.58, 12.02, 8.14, 18.50, 8.45-fold higher cytotoxicity in 8226, KM3, U266, ARP-1, and MM1.s cells, respectively (Figure 6D).

Next, we performed Western blot and MTT assay to determine whether WBC158 inhibited the growth of MM cells partially by targeting CAMKIIγ. First, we treated U266 and KM3 cells with different concentrations of WBC158 for 72h. We found that the levels of activated CAMKIIγ (p-CAMKIIγ) and p-Stat3 but not total CAMKIIγ in both U266 and KM3 cells decreased in dose-dependent manners. (Figure 6E). To confirm whether CAMKIIγ is an important target of BBM and WBC158, we performed CAMKIIγ-knockout (CAMKIIγ-KO) in U266 cells (Figure 6F), and then determined effect of BBM and WBC158 on viability of U266 cells. We observed that CAMKIIγ-KO conferred 1.44-fold and 1.55-fold decreases, respectively, in sensitivity to BBM and WBC158 (Figure 6G and 6H). These results strongly suggest a direct relationship of CAMKIIγ levels with BBM and WBC158 response.

**Figure 7:**
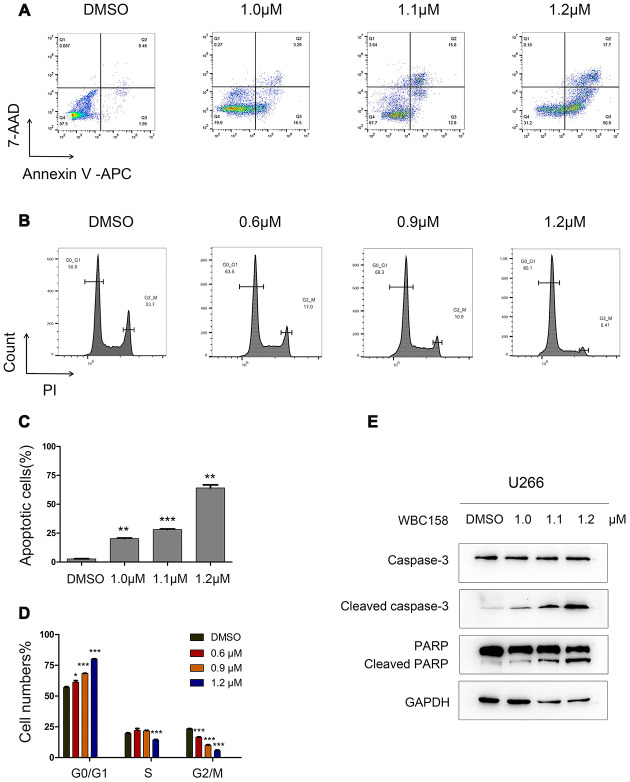
**WBC158 induced apoptosis of MM cells and retarded cell cycle progression.** U266 cells were incubated with WBC158 at the indicated concentrations for 24h. Representative images of apoptosis (**A**) and cell cycle (**B**). Statistical graphs of apoptosis (**C**, **P < 0.01, ***P < 0.001) and cell cycle (**D**, *P < 0.05, ***P < 0.001). (**E**) Western blot of apoptosis-related proteins.

To detect whether WBC158 played a role in apoptosis and cell cycle of MM cells, we treated U266 cells with WBC158 at different concentrations for 24h. As a consequence, WBC158 potently induced apoptosis (Figure 7A, 7C) and suppressed cell cycle progression (Figure 7B, 7D). Consistent with these results, apoptosis-associated protein cleaved-Caspase 3 and cleaved-PARP increased in dose-dependent manners in U266 cells after treatment with WBC158 for 24h (Figure 7E). Cell cycle analysis showed WBC158 treatment resulted in increased of G0/1 cells and decreases of S and G2/M cells in U266 cells (Figure 7D). These results indicate that WBC158 is new potent agent that induces apoptosis of MM cells through targeting CAMKIIγ.

### BBM and its novel analogue WBC158 suppress the growth of CAMKIIγ-dependent MM in vivo

**Figure 8:**
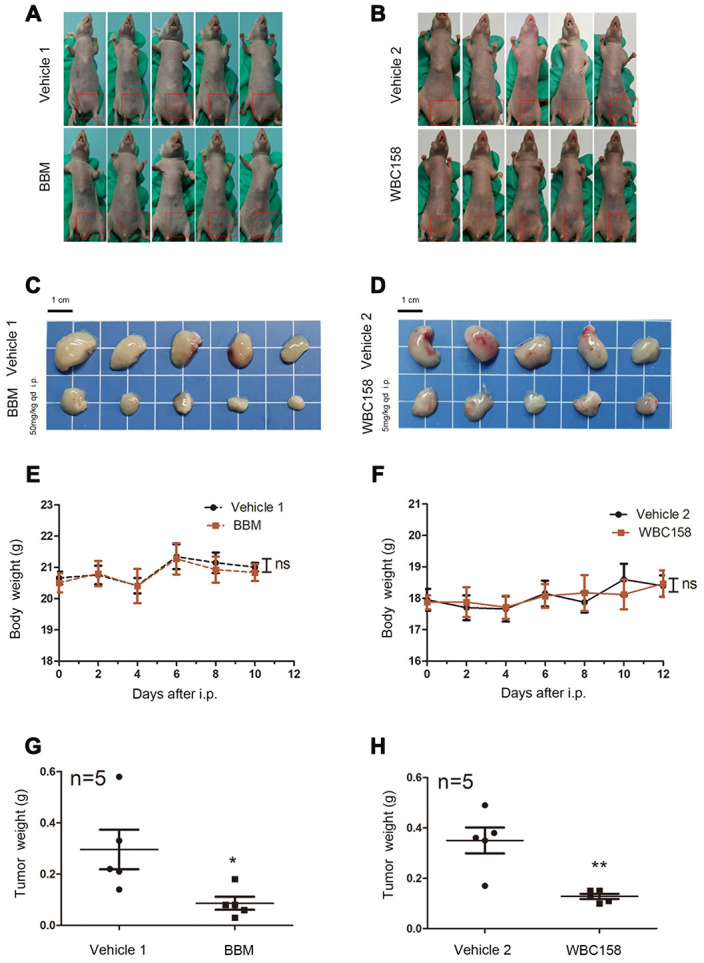
**BBM and WBC158 inhibited the growth of CAMKIIγ-dependent MM in vivo.** (**A**, **B**) U266 cells were subcutaneously injected into the left groin of nude mice. Mice treated with BBM or WBC158 had smaller tumors than their controls after nearly three weeks. (**C**, **D**) Images of xenograft tumors from the indicated groups. (**E**, **F**) Body weight of nude mice (no significance). (**G**, **H**) Tumor weight (*P < 0.05, **P <0.01).

To assess in vivo the effect of BBM and its analogue WBC158 on CaMKIIγ-dependent multiple myeloma, we first established human MM xenograft model using nude mice with CaMKIIγ-dependent U266 cells, which were implanted subcutaneously into the groin region of nude mice (Figure 8A, 8B). Consistent with the in vitro results, intraperitoneally administered BBM and WBC158 exerted anti-tumor activity in human CaMKIIγ-dependent U266 cells in nude mice (Figure 8C, 8D). Treatment with 50 mg kg-1dose of BBM and 5 mg kg-1 dose of WBC158 resulted in 70.95%, and 63.43% of tumor growth inhibition (TGI), respectively (Figure 8G, 8H). Notably, BBM and WBC158 were well tolerated by the mice with no significant body weight loss observed (Figure 8E, 8F). These results suggest that targeting CaMKIIγ by BBM analoges has potential to regress CaMKIIγ-dependent MM.

## DISCUSSION

Despite the application of PI, IMiDs, monoclonal antibodies and stem-cell transplantation, MM retains its tendency to develop drug resistance, which leads to recurrence and poor outcome in clinical treatment. So, here is an urgent need to search for other specific new therapeutic targets and alternative drugs for treating cancer.

CAMKIIγ, a critical regulator of tumor formation, can facilitate tumor cell proliferation, malignant development and unfavourable prognosis [9, 23–28]. Our previous studies demonstrated that CAMKIIγ can activate multiple leukemia-associated signaling networks by NF-κB, β-catenin and Stat3 pathways, which are essential for survival and self-renewal of leukemia stem cells (LSC) [11]. In this study, we found that expression levels of CAMK2G were higher in MM patients than those in healthy donors, and was statistically upregulated at advanced stage, compared to stage I, through analyzing the data from GEO database. Consistently, we observed that 36 of 53 MM samples showed medium to strong expression of CAMKIIγ, and high expression of CAMKIIγ accompanied with worse clinical outcomes, suggesting that CAMKIIγ may play an important role in MM. Importantly, we further demonstrated that CAMKIIγ is essential for the survival and proliferation of MM cells and CAMKIIγ overexpression significantly promoted tumor cell growth and colony formation in vitro and in vivo.

Mechanically, our studies reveal that upregulation of CAMKIIγ promotes cell cycle of MM cells, whereas downregulation of CAMKIIγ induces G0/1 phase arrest, G2/M phase decrease and apoptosis of U266 cells, suggesting that CAMKIIγ plays an important role in survival and proliferation of MM cells. Moreover, we demonstrate that CAMKIIγ is critically required for activation of ERK and Stat3 in MM cells. It is worth mentioning that MAPK/ERK and Stat3 are identified as therapeutic targets for MM [29, 30–32]. For instance, Stat3 activation is correlated with adverse prognostic and may promote drug resistance among MM patients [11, 32–35].

Our previous studies showed that BBM is a specific CAMKIIγ inhibitor and suppressed leukemia, lymphoma and solid tumors with high dose [9, 11, 36] suggesting that it could be used as an effective compound for developing pharmacological small molecule inhibitor of CAMKIIγ. In this study, our data not only showed that BBM could potently inhibit the growth of human CAMKIIγ-dependent MM xenografts in mouse model but also demonstrated that the novel BBM derivative WBC158 has more potent anti-tumor activity in vitro and in vivo as compared with BBM.

In summary, we for the first time demonstrate that CAMKIIγ plays a critical role in maintaining MM cell growth through upregulating STAT3 signaling pathway and BBM analogues might be new small molecule inhibitors for treating CAMKIIγ-dependent MM.

## MATERIALS AND METHODS

### Data preparation

The publicly available datasets of mRNA-expression in the study include the datasets under the NCBI Gene Expression Omnibus (GEO) accession numbers GSE5900 and GSE13591. These microarray datasets were downloaded and then statistical significance was calculated by Student’s t-test with SPSS 25.0 software.

### Patients and tissue samples

We collected formalin-fixed paraffin-embedded (FFPE) tissue samples, which were focally infiltrated by myeloma cells, of 53 untreated MM patients with extramedullary disease diagnosed between May, 2007 and June, 2015 in the Second Affiliated Hospital, Zhejiang University School of Medicine (Zhejiang, China) (#IR2019001647). In total, the patient tissue samples were consisted of 51 bone tissues and 2 soft tissues. The clinical data were obtained from hospital medical record and follow-up information were collected through telephone calls. This study was approved by the Ethics Committee of the Second Affiliated Hospital, Zhejiang University School of Medicine.

### Antibodies

Phospho-CAMKII (Thr287) and CAMKIIγ antibodies (#sc-517278) were purchased from Santa Cruz Biotechnology (Santa Cruz, USA). CAMKIIγ antibody (#12666-2-AP) was obtained from Proteintech (USA). GAPDH antibody was from Huabio (China) and Syndecan-1 (CD138) antibody was from Bioss Antibodies (China). PARP, Caspase-3, Cleaved Caspase-3, LC3B, p44/42 MAPK (ERK1/2), phospho-p44/42 MAPK (ERK1/2), phospho-JAK2 (Tyr1007/1008), JAK2, phospho-Stat3 (Tyr705), and Stat3 antibodies were obtained from the Cell Signaling Technology (CST, USA). Flag was purchased from ABclonal (USA).

### Immunohistochemistry (IHC) analysis

Each sample was fixed with 4% buffered paraformaldehyde, embedded in paraffin, and then cut into 4 μm sections. Subsequently, the deparaffinized sections were performed with PH9.0 Tris-EDTA boiling in a microwave for antigen retrieval. These sections were incubated with CAMKIIγ antibody (#12666-2-AP) overnight at 4°C. Secondary antibody was incubated with the slides at room temperature for 30 minutes.

H&E and CD138 immunostain of serial sections were obtained from the department of pathology. The staining density and positive staining rate of each slide was evaluated by an experienced pathologist. The quantity score evaluated the positive proportion of plasma cells for CAMKIIγ protein was recorded as 0 (< 5% labelled cells), 1 (5% to 25% labelled cells), 2 (26% to 50% labelled cells), 3 (51% to 75% labelled cells), 4 (> 75% labelled cells). The intensity of immunostaining was scored as 0 (no immunostaining), 1 (weakly immunostaining), 2 (moderately immunostaining) and 3 (strongly immunostaining). A final score was calculated by multiplying the staining extent and intensity scores. Final score < 8 was defined as low CAMKIIγ expression, and high expression with a final score ≥ 8.

### Immunofluorescence histochemistry (IF)

Tissue samples were blocked with blocking buffer for 1 hour, and then incubated with the CAMKIIγ antibody (#sc-517278) and CD138 antibody overnight at 4 °C. After washing three times with PH 7.4 phosphate-buffered saline (PBS), the samples were incubated with the secondary antibodies for 3 hours at room temperature. Images were acquired under a Zeiss Confocal Laser Scanning Microscope 710 (LSM710, Germany).

### Cell lines and cell culture

U266, RPMI8226, KM3 and 293T cell lines were obtained from the Cancer Institute of Zhejiang University, China. MM1.s and ARP-1 cell lines were kindly provided by Prof. Zhen Cai (Zhejing University, China). 293T cells were cultured in DMEM medium (Gibco, USA) supplemented with 10% fetal bovine serum (FBS, Gibco), 100 μg/ml streptomycin and 100 U/mL penicillin (Gibco). Human MM cell lines were maintained in RPMI1640 medium (Gibco) with 10% fetal bovine serum (FBS, Gibco), 100 μg/ml streptomycin and 100 U/mL penicillin (Gibco). Cells were cultured at 37°C in a humidified 95% air, 5% CO2 incubator (Herea, USA).

### Plasmids

The human CaMK2G coding sequence (NM_172171.2) with a 3×FLAG sequence and a kozak sequence was cloned into the vector pCDH-MSCV-MCS-EF1α-GFP+Puro (System Biosciences, USA) using Hieff Clone TM One Step Cloning Kit (Yeasen, China) [26].

CRISPR/Cas9 system was applied to construct cells with CaMK2G knockout. Lentivirus vector pCW-Cas9 (doxycycline inducible; #5066) and pLX-sgRNA (lentiviral vector, #50662) were purchased from Addgene (USA). Two seperate sgRNAs against CaMK2G were designed and cloned into pLX-sgRNA following the the CRISPR protocol [37]. The sequence of two seperate sgRNA against CaMK2G as follows: 5’-CACCGTGCTTTCTCTGTGGTCCGC-3’; 5’-AAACGCGGACCACAGAGAAAGCAC-3’.

### Lentiviral preparation, infection and stable cell generation

293T cells were cotransfected with viral packaging vectors psPAX2 and pMD2.G (Addgene, USA), along with the lentiviral construct expressing vector or the empty vector as control. Polyjet transfection reagent (SignaGen, USA) was supplemented in the medium to improve the transfection efficiency. The virus supernatant was collected after 48 hours of transfection, and concentrated by ultracentrifugation at 4,000 g for 20 minutes. Generation of lentivirus as required was performed as described. Then, cells were incubated with supernatant and polybrene (Gene, China) supplemented in the cell growth medium.

For construction of CAMK2G overexpression cells, KM3 and U266 cells were transfected with CAMK2G over-expression lentivirus or the empty vector as control. After 24 hours, the medium was replaced; 72 hours later, puromycin (2 μg/ml) was added to the medium for selecting the transfected cells.

To generate CAMK2G-knockout cells, U266 cells were transfected with pCW-Cas9 lentivirus and then selected with Blasticidin S (400 μg/mL) for two weeks. After Cas9-Flag protein was detected by western blot, the cells were transfected with CAMK2G sgRNA lentivirus or sgRNA lentivirus as control. Later, puromycin (2 μg/ml) was added to the infected cells for selection.

### Cell survival/proliferation assay

MM cell lines (3-10 x 10^4 per well) were seeded into 96-well plates with 200 μL medium, cultured for 72h in the presence of required concentrations of BBM analogues. Especially, the medium of U266-transfected cell lines were required to contain Dox (2 μg/ml). After 4h of incubation with MTT, acidified SDS was added to each well overnight to dissolve the purple formazan produced by the viable cells and MTT. The higher absorbance suggested higher cell viability and IC_50_ was regarded as the drug concentration that could decrease 50% cell viability.

### Apoptosis assay, cell cycle assay and Flow cytometry analysis

Apoptosis was evaluated by Annexin V-APC/7-AAD Apoptosis Kit (MULTISCIENCES, China) and cell cycle was measured by Cell cycle staining Kit (MULTISCIENCES, China) according to the manufacture’s protocol. The analysis was performed on CytoFLEX LX Flow Cytometer (Beckman Coulter, USA) and the data was processed by FlowJo V software.

### Western blot analysis

The total cellular protein was extracted using M-PER Mammalian Protein Extaction Reagent supplemented with 1% protease and phosphatase inhitor (Thermo Scientific, USA). Proteins were separated by SDS-PAGE, transferred to PVDF membranes (Bio-Rad, USA) and blocked with 5% non-fat milk in TBST buffer (TBS-Tween 20). Then membranes were incubated with primary antibodies overnight at 4°C. After washing 3 times with TBST, membranes were treated with horseradish peroxidase-conjugated secondary antibodies (Huabio, China) for 1 hour at room temperature and probed with SuperSignal West Pico Chemiluminescent Substrate (Thermo Scientific, USA).

### Soft agar clony-formation assays

U266 transfected cells (800 cells per well) or KM3 transfected cells (200 cells per well) were suspended in 1.5 mL of 2x1640 containing 20% FBS with 1.5mL of 0.7% low-melting-point agarose (BBI Life Sciences, China) in sterile water. Cells were dispensed to 6-well plates and layered on top of 0.6% agarose with 10% FBS at 3mL per well in triplicates. Particularly, the medium for U266 transfected cell lines was allowed to contain Dox (2 μg/ml). After roughly 3 weeks, the colonies were stained with crystal violet, then scored and photographed.

### In vivo studies

BALB/c female nude mice of age (6-7 weeks) and weight (18-21 g) were purchased from the SLAC (Shanghai, China) and the SPF Biotechnology (Beijing, China), which was kept under specific pathogen-free (SPF) conditions in the animal laboratory of the Second Affiliated Hospital, Zhejiang University School of Medicine. All experimental procedures were approved by the Ethics Committee of the Second Affiliated Hospital Zhejiang University School of Medicine (#2019-082).

To establish xenograft model, cells were subcutaneously injected (KM3 control, KM3 CAMKIIγ; U266 control, U266 CAMKIIγ KO: 1.5x10^7 cells in 0.2 mL PBS per site) into the left groin of nude mice. After the development of palpable tumors, mice in groups of U266 transfected cells would receive 0.4 mL DOX (10mg/ml) to initiate CAMKIIγ silencing via oral gavage once a day. The mice were sacrificed and the subcutaneous tumors were dissected out, when the largest tumor within the same group reached 1.5 cm in diameter.

BBM and Citric acid (DAMAO, China) in equimolar amounts were dissolved in PBS (TBD, China) at a concentration of 5mg/ml, and WBC158 (Weben Pharmaceuticals) with the final concentration of 0.25 mg/ml. The vehicles had the same concentration of citric acid dissolved in PBS (vehicle 1, vehicle2). To evaluate the role of BBM analogues in MM murine xenograft model, nude mice were injected subcutaneously in the left groin with 1.5x10^7 U266 cells in 0.2 mL PBS. When palpable tumors became detectable, approximately a week after injection of U266 cells, the mice were randomly divided into different groups to receive vehicles (Control groups), BBM (50 mg/kg, BBM group) or WBC158 (5 mg/kg, WBC158 group) treatment via intraperitoneal injection once a day for nearly 12 consecutive days, respectively. We defined the first day of treatment as Day 0 and started to record body weight every other day.

### Statistical analysis

Statistical analyses were performed with SPSS 25.0 and GraphPad Prism 5.0 software. All data were expressed as the mean ± SEM and showed error bars. Statistical significance was calculated by Student’s t-test, two-way ANOVA, Mann-Whitney U test, Chi-square test, log-rank test as indicated. *P* value < 0.05 was considered to be statistically significant (*P < 0.05; **P < 0.01; ***P < 0.001).
